# High-fat diet intensifies *MLL-AF9*-induced acute myeloid leukemia through activation of the FLT3 signaling in mouse primitive hematopoietic cells

**DOI:** 10.1038/s41598-020-73020-4

**Published:** 2020-09-30

**Authors:** François Hermetet, Rony Mshaik, John Simonet, Patrick Callier, Laurent Delva, Ronan Quéré

**Affiliations:** 1 Inserm UMR1231, Université Bourgogne Franche-Comté, Dijon, France; 2 LipSTIC LabEx, Dijon, France; 3 Laboratoire de Génétique Chromosomique et Moléculaire, Plateau Technique de Biologie, CHU Dijon, Dijon, France

**Keywords:** Cancer, Cancer models

## Abstract

Using a *MLL-AF9 knock-in* mouse model, we discovered that consumption of a high-fat diet (HFD) accelerates the risk of developing acute myeloid leukemia (AML). This regimen increases the clusterization of FLT3 within lipid rafts on the cell surface of primitive hematopoietic cells, which overactivates this receptor as well as the downstream JAK/STAT signaling known to enhance the transformation of *MLL-AF9 knock-in* cells. Treatment of mice on a HFD with Quizartinib, a potent inhibitor of FLT3 phosphorylation, inhibits the JAK3/STAT3, signaling and finally antagonizes the accelerated development of AML that occurred following the HFD regimen. We can therefore conclude that, on a mouse model of AML, a HFD enforces the FLT3 signaling pathway on primitive hematopoietic cells and, in turn, improves the oncogenic transformation of *MLL-AF9 knock-in* cells and the leukemia initiation.

## Introduction

The obesity-related cancer burden represents up to 9% of all cancer cases^1^. Even if major role has been established for high-fat diet (HFD) in several solid cancers (e.g. gastric cardia, colon, rectum and liver cancer)^2^, only a small number of epidemiological studies have been conducted on leukemia. A study provides the evidence for an association between a Western dietary pattern and chronic lymphocytic leukemia, suggesting that a proportion of cases could be prevented for this disease by modifying dietary habits^3^. Among hematological diseases, diet-induced obesity has been shown related to myeloma development^4^. Although acute myeloid leukemia (AML) is a relatively rare disease, accounting for roughly 1.2% of cancer deaths, its incidence should increase as the population ages. In a cohort of more than half a million individuals, those eating large quantities of food were more likely to develop AML^5^. In addition, a higher body mass index is associated with poorer survival in pediatric AML^6^. The leukemia burden of AML is also much higher in HFD-induced obese mice^7,8^.

We and others have already shown that HFD induces major perturbations in murine hematopoietic cells, such as a loss of the hematopoietic stem cells (HSC), as well as in the homeostasis of the mouse hematopoietic system^9–15^. The role of HFD on the promotion of leukemia is however still poorly explained, the data available are sparse concerning HFD impact on disease initiation and development in AML, and there is no study, to the best of our knowledge, on the HFD potential mechanism of action leading to the transformation of normal primitive hematopoietic cells into AML cells.

In this study, we therefore investigated whether a HFD could accelerate the risk of developing AML. The *MLL-AF9 knock-in* mouse model reflects aspects of AML in humans and it can be used to provide biological insights into MLL-rearranged leukemogenesis^16,17^. The purpose of this study was first to describe how feeding mice a HFD over a short period influences the initiation and development of AML and then to characterize how the consumption of a HFD transforms primitive hematopoietic cells.

## Materials and methods

### Use of experimental animals

The ethics committee for animal welfare of the University and the French ministry of higher education and research approved all animal experiments (under reference APAFIS#16187-2018071914379464v3). We confirm that all experiments were performed in accordance with relevant guidelines and regulations of this committee.

### Mice

*MLL-AF9* (Kmt2a<tm2(MLLT3)Thr>/KsyJ, Jackson Laboratory), C57bl/6J (Charles River and Envigo) and C57bl/6.SJL (Ly.1) mice (Charles River) were kept in the animal facility at the University of Burgundy. Genotyping of *MLL-AF9 knock-in* mice was done following a protocol described by the manufacturer online (https://www.jax.org/strain/009079). Mice were maintained on a rodent chow diet ad libitum, composed of 4% fat for the control diet (CD) or 42% fat for the high-fat diet (HFD; MD. 88137, Envigo, France). Quizartinib (SelleckChem) was reconstituted at 10 mg/mL in Dimethyl Sulfoxyde (DMSO) and injected in the peritoneum at 5 mg/kg, twice per week, during the 4 weeks of HFD feeding.

### Transplantations

The BM cells (2 × 10^5^ cells) isolated from *MLL-AF9 knock-in* mice were transplanted into the tail veins of lethally irradiated (900 cGy, Biomep, Dijon, France) C57bl/6J recipient mice. Thirty days after transplantation, the mice were divided into two groups: one was fed a HFD for 4 weeks and the other was fed a CD. After 4 weeks of a HFD, the mice were fed a CD ad libitum. For the cell-transforming potential (CTP) study, mice were transplanted with 2 × 10^3^, 2 × 10^4^ or 2 × 10^5^ cells isolated from *MLL-AF9 knock-in* mice together with 2 × 10^5^ Ly.1 supported cells isolated from the BM of a wild-type C57bl/6J mouse. We analyzed AML development in lethally irradiated recipient Ly.1 mice, by transplantation of 2 × 10^4^ AML cells with 2 × 10^5^ Ly.1 supporter cells.

### Peripheral blood (PB) and bone marrow (BM) cells analyses

After tail vein PB sampling, white blood cells (WBC) were counted with a hemocytometer (SCIL Vet ABC^+^, Oostelbeers, The Netherlands). PB was monitored monthly for occurrence of AML. Bones were crushed in a mortar and total BM cells were filtered (30 µm).

### Flow cytometry and FACS

For staining of HSC and progenitors, we used antibodies and strategies for gating as previously described^10^. For western blot and immunostaining, c-KIT^+^ cells were isolated from BM after 4 weeks of CD or HFD with magnetic murine CD117-Microbeads (Miltenyi Biotec). Magnetically lineage-depleted (Lin^−^) BM cells (Miltenyi Biotec) were stained in PBS 1x with combinations of antibodies. We used CD45.2-PE-Cy7, c-KIT-Pacific Blue (PB), SCA1-APC-Cy7, FLT3 (CD135)-PE, CD34-AF647, CD16/32-FITC and IL7Rα-PE-CF594 on Lin^−^ cells, 4-weeks after the CD or HFD. To analyze phenotype of AML, MAC1-AF647 and GR1-FITC (BD Biosciences) were used on total BM cells, when mice have developed leukemia. We used KI67-FITC (BD Biosciences) and propidium iodide (BD Biosciences) on AML cells isolated ex vivo from BM and permeabilized for intracellular staining (BD Cytofix/Cytoperm, BD Pharmingen). Cell subsets were analyzed using a FACS Canto10 or a LSR-Fortessa (BD Biosciences) and sorted on a FACSAriaIII cell sorter (BD Biosciences). Data were analyzed using FlowJo software (version 10, TreeStar Inc, https://www.flowjo.com).

### Western blot

Western blot was performed on c-KIT^+^ cells isolated with magnetic murine CD117-Microbeads (130-091-224, Miltenyi Biotec) from BM after 4 weeks of CD or HFD. We used antibodies to detect FLT3 (PA5-34448, Thermo Fisher Scientific) or ACTB (612656, BD Biosciences). We used JAK3 (#8863), phospho-JAK3 (Y980/981) (#5031), STAT5 (#94205), phospho-STAT5 (Y694) (#9359), STAT3 (#4904), phospho-STAT3 (Y705) (#9145) antibodies, all from Cell Signaling. After immunoprecipitation of all tyrosine phosphorylated proteins with an anti-phosphotyrosine antibody (05-321, Millipore), FLT3 was analyzed by western blot (PA5-34448, Thermo Fisher Scientific). After immunoprecipitation with an anti-FLT3 antibody (PA5-34448, Thermo Fisher Scientific), phosphorylation of FLT3 was assessed with an anti-phosphotyrosine antibody (05-321, Millipore). Gel images were processed and analyzed for quantification (Fiji, NIH).

### Immunofluorescence and microscopy

After 4 weeks of the CD or HFD, c-KIT^+^ cells were isolated with magnetic CD117-Microbeads (Miltenyi Biotec) from BM. Cells were stained with rabbit anti-FLT3 (PA5-34448, Thermo Fisher scientific) and secondary anti-rabbit-AF488 antibodies (Thermo Fisher Scientific). Lipid rafts were stained with cholera toxin subunit B conjugated with AF555 (Thermo Fisher Scientific). Cells were placed on glass slides for 5 min. ProLong Gold Antifade Mountant containing DAPI (Thermo Fisher Scientific) was applied directly to fluorescently labeled cells on microscope slides. Fluorescence was observed by microscopy (Axio Imager 2, Zeiss) and the images were processed (Fiji, NIH).

### Reverse transcription quantitative polymerase chain (RTqPCR) reaction

After mRNA isolation with the RNeasy kit (Qiagen), M-MLV reverse transcriptase (Promega) was used to synthesize cDNA. The following TaqMan assays were then used for qPCR: *Flt3* (Mm00439016) and *Hprt1* (Mm03024075) used as endogenous controls. We used GoTaq Probe qPCR Master Mix (Promega). Experiments were carried out using the Viia7 system (Applied Biosystems).

### DNA sequencing

Genomic DNA from AML cells isolated from the BM of 8 mice (from CD and HFD groups) that developed leukemia was extracted after cell lysis and protein precipitation (Qiagen); DNA was then precipitated with ethanol. PCR was performed with the following primers; *Flt3-e14-15-F*: TGCGACCATTGGGCTCTGTCTCCCCTTC; *Flt3-e14-15-R*: ACTGGC-CCTGACAGTGTGCATGCCCCC; *Flt3-e20-F*: GAGGAGGAAGATTTGAA-CGTGCTGACG; *Flt3-e20-R*: CCAGAGAAGGATGCCGTAGGACCAGACG. Sequencing was performed by Sanger (Genewiz).

### CGH array

For the Array Comparative Genomic Hybridization (CGH array), we used the SurePrint G3 Mouse CGH Microarray Kit, 4 × 180 K (Agilent Technologies) and 1 µg of genomic DNA extracted from AML samples using Gentra Puregene Tissue kit (Qiagen). For analysis, we used the G2505 DNA microarray Scanner (Agilent Technologies) and the Agilent Cytogenomics software was used (version 2.7, Agilent Technologies, https://www.agilent.com/en/download-agilent-cytogenomics-software).

### Statistics

All data were expressed as means ± SD or presented as median, first and third quartiles, and whiskers. Differences between groups were assessed with the Student’s unpaired t-test. Statistical analysis of survival curves were assessed using the Mantel–Haenszel Logrank test. Statistics were performed using Prism 6 (GraphPad), significance are indicated on the figures with the following convention: *, *P* < 0.05; **, *P* < 0.01; ***, *P* < 0.001.

### Ethical standards

The ethics committee for animal welfare of the University and the French ministry of higher education and research approved all animal experiments (under reference APAFIS#16,187-2018071914379464v3). We confirm that all experiments were performed in accordance with relevant guidelines and regulations of this committee.

## Results

### HFD increases the risk of AML development in mice

**Figure 1:**
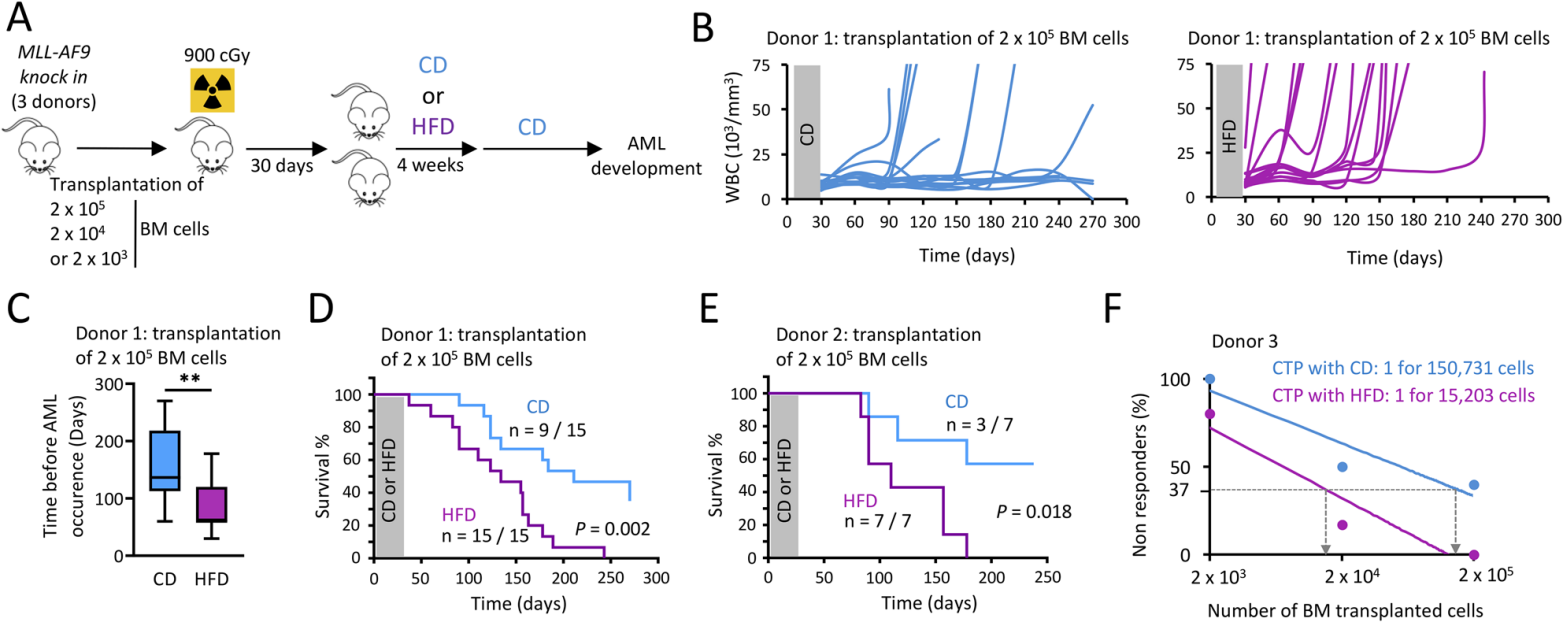
HFD increases the risk of AML development in mice. (**A**) Experimental workflow describing the procedure. Image performed with the GIMP software (version 2.10.18, GIMP, https://www.gimp.org/news/2020/02/24/gimp-2-10-18-released/). (**B**) Level of white blood cells (WBC) over time in the peripheral blood (PB) of mice fed with either a CD or a HFD. Mice fed a HFD developed more AML than CD-fed mice. PB was monitored monthly for occurrence of AML, n = 15 mice per group. (**C**) Time before observing AML occurrence following the BM cell transplantation, showing that this period is reduced for HFD-fed mice compared to CD-fed mice. We considered mice start to develop AML, when the WBC is over 15 × 10^3^/mm^3^ in PB. Data are presented as median (central line), first and third quartiles (bottom and top of boxes, respectively), and whiskers (extreme values); n = 9 and 15 mice for CD and HFD-groups, respectively. **P < 0.01, two-tailed unpaired Student’s t-test. (**D**) Survival curves showing that mice fed a HFD developed more AML than CD-fed mice, n = 15 mice per group; *P* value measured by Mantel–Haenszel test. (**E**) Survival curves with another *MLL-AF9 knock in* donor, n = 7 mice per group; *P* value measured by Mantel–Haenszel test. (**F**) Transplantation of varying numbers of *MLL-AF9 knock in* donor cells showing that the number of cell-transforming potential (CTP) increased when mice were fed a HFD. Transplantation of 2 × 10^3^ cells, n = 5 mice; 2 × 10^4^ cells, n = 5 mice; 2 × 10^5^ cells, n = 5 mice.

We transplanted bone marrow (BM) cells from a *MLL-AF9 knock-in* mouse (Ly.2) into lethally irradiated C57bl/6.SJL (Ly.1) recipient mice that were divided in two groups: over a period of 4 weeks, one group was fed a control diet (CD) and the other a HFD (Fig. 1A). The HFD did not alter engraftment of *MLL-AF9 knock-in* Ly.2 BM cells to the BM, or the reconstitution of total white blood cells (WBC) in the peripheral blood (PB) of recipient mice (Supplementary Fig. S1). As we previously described in mice fed a 4-week HFD^10^, among lineage negative (Lin^−^) cells, we observed a decrease of the primitive HSC population (SCA1^+^ c-KIT^+^ CD34^−^), but there was no impact on the distribution of other mature progenitors such as mega-erythroid progenitor (MEP), common myeloid progenitor (CMP), granulocyte/macrophage progenitor (GMP), and common lymphoid progenitor (CLP) (Supplementary Fig. S2).

HFD-fed mice developed AML much more quickly than did mice from the control group, as assessed by the increase in the total WBC count in the PB for HFD-fed mice (Fig. 1B), and times to AML occurrence (*P* = 0.006, Fig. 1C) and to death following AML development (*P* = 0.002, Fig. 1D). Using BM cells from another *MLL-AF9 knock-in* mouse donor, we confirmed that HFD accelerates development of AML (*P* = 0.018, Fig. 1E). Furthermore, after we transplanted different numbers of *MLL-AF9 knock-in* BM cells into lethally irradiated recipient mice, we determined that the risk for primitive hematopoietic stem/progenitor cells (HSPC) of being transformed into leukemia stem cells, mentioned as cell-transforming potential (CTP), was tenfold higher when mice consumed a HFD (Fig. 1F).

We can therefore conclude that HFD has intensified *MLL-AF9*-induced AML.

### HFD did not modify the phenotype of resulting transformed AML cells

**Figure 2:**
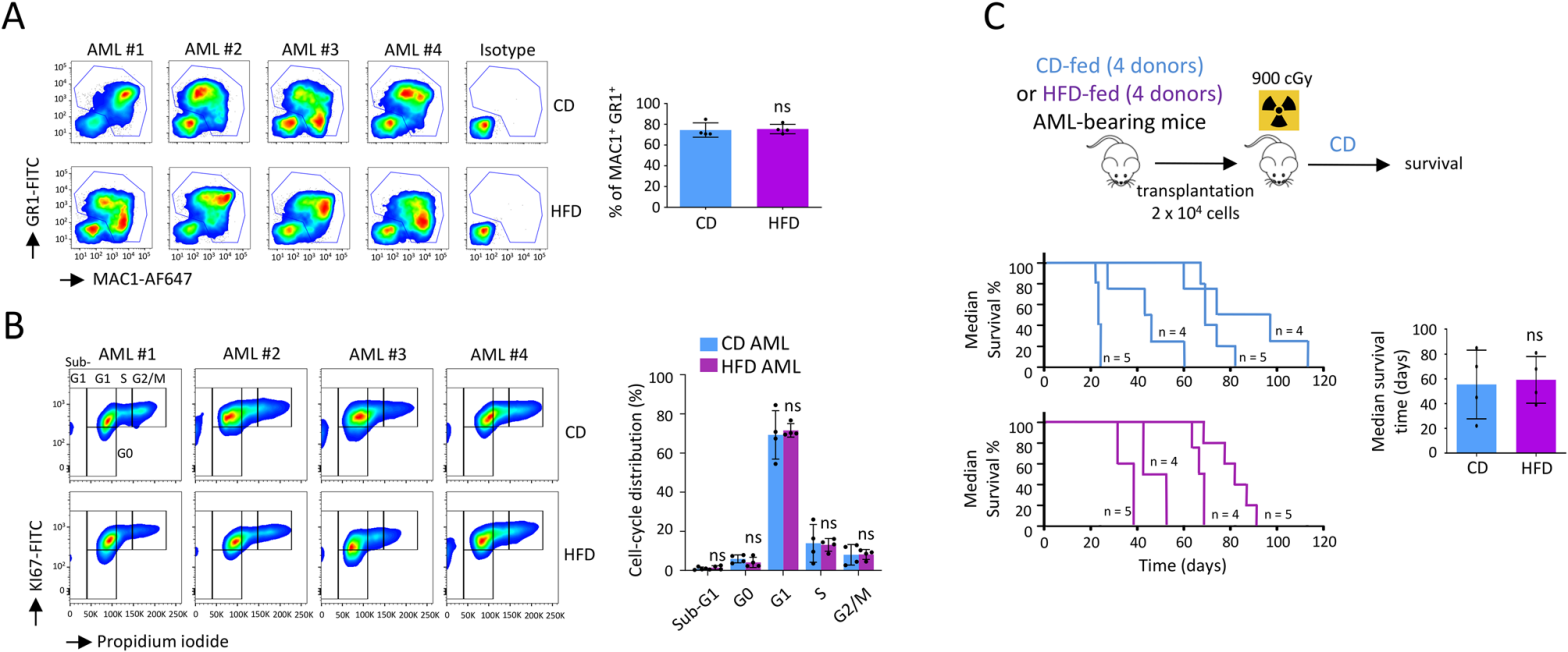
HFD did not modify the phenotype of resulting transformed AML cells. (**A**) AML cells isolated from BM expressed MAC1 and GR1 myeloid markers, with no difference observed between CD- and HFD-fed mice. Flow cytometry showing expression of MAC1 and GR1 and statistic of the percentage of leukemic cells in BM. Data show mean ± SD; n = 4 mice (#1–4) per diet group; *P* value measured by two-tailed unpaired Student’s t-test; ns, non-significant. Data were analyzed using FlowJo software (version 10, TreeStar Inc, https://www.flowjo.com). (**B**) Cell cycle study performed by flow cytometry on AML cells isolated from BM of CD- and HFD-fed mice. Data show mean ± SD; n = 4 mice (#1–4) per diet group; *P* value measured by two-tailed unpaired Student’s t-test; ns, non-significant. Data were analyzed using FlowJo software (version 10, TreeStar Inc, https://www.flowjo.com). (**C**) Post-CD and -HFD AML cells are similarly aggressive in vivo. Experimental workflow describing the procedure. Image performed with the GIMP software (version 2.10.18, GIMP, https://www.gimp.org/news/2020/02/24/gimp-2-10-18-released/). Survival curves after the transplantation of 2 × 10^4^ AML cells isolated from 4 different AML-bearing mice from the CD and HFD groups (n = 4 samples analyzed in each group). Recipient mice (n = 4 or 5 transplanted mice with each samples of initial post-CD and -HFD AML cells) were fed a CD and monitored for occurrence of AML. Median survival time was calculated, n = 4 AML per diet group; *P* value measured by two-tailed unpaired Student’s t-test; ns, non-significant.

AML cells expressed MAC1 and GR1 myeloid markers, with no difference observed between CD- and HFD-fed mice (Fig. 2A). AML cells extracted ex vivo from BM were also active, as assessed by equivalent cell cycling activities measured by flow cytometry, after KI67 and propidium iodide staining (Fig. 2B). CGH-array showed no genetic alteration in DNA isolated from post-HFD AML cells, compared with post-CD AML samples (Supplementary Fig. S3). To check if the HFD induced leukemia with different levels of aggressiveness, we transplanted AML cells from primary mice into lethally irradiated C57bl/6.SJL (Ly.1) recipient CD-fed mice and then monitored for occurrence of AML. In these host mice transplanted with 2 × 10^4^ AML cells from both CD and HFD-fed mice, we observed variability between AML samples to generate leukemia, without any significant difference between diet groups. Indeed, this experiment suggested that there is no variability in leukemia initiating cell (LIC) frequency between post-CD and -HFD AML samples (Fig. 2C).

We can therefore conclude that a HFD did not modify the phenotype of the resulting AML cells, their cell cycling activities, or their capacities to engraft and generate leukemia in recipient mice fed a CD.

### HFD activates the FLT3/JAK3/STAT3 signaling on primitive murine hematopoietic stem/progenitor cells

**Figure 3:**
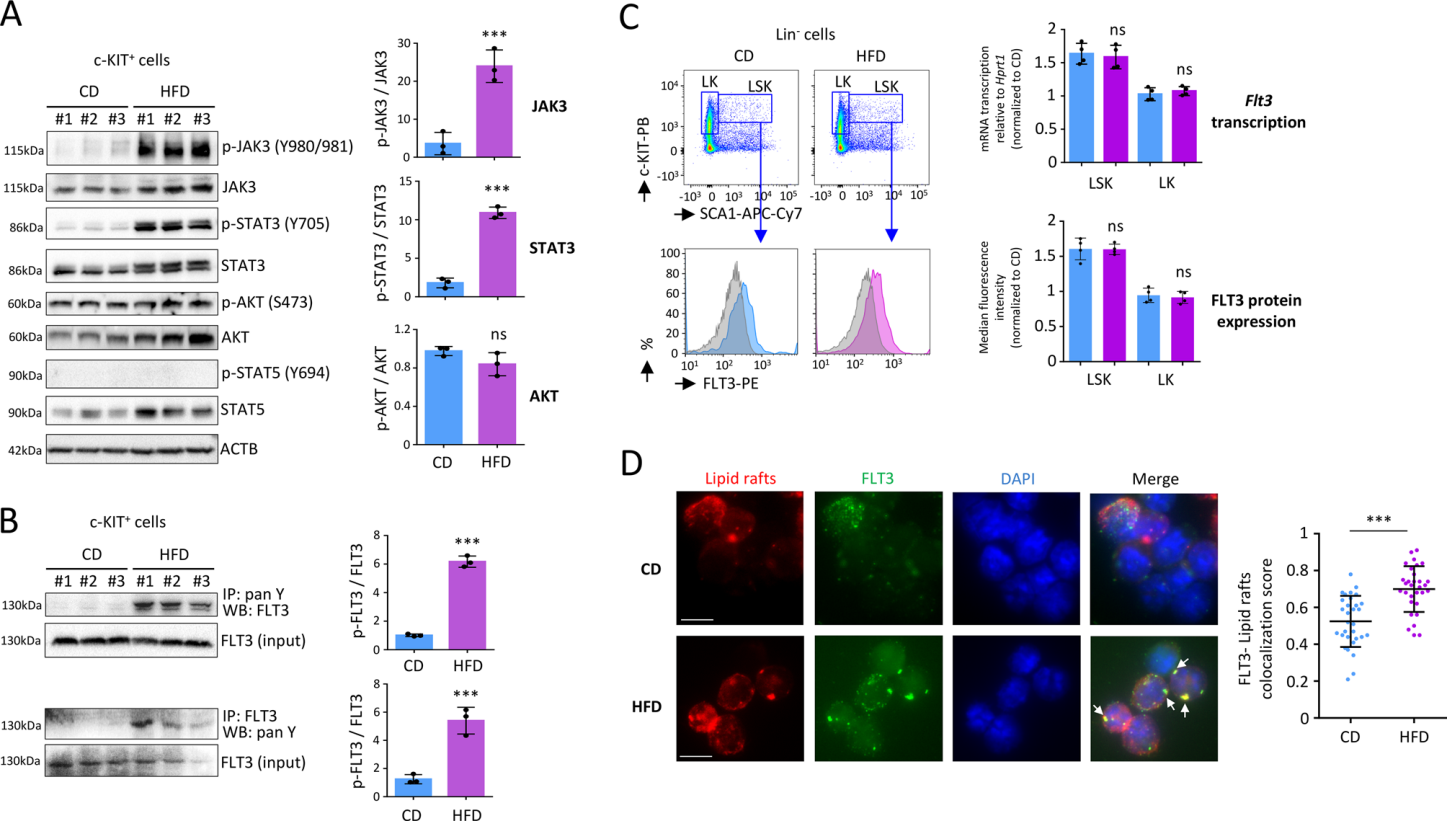
HFD activates FLT3/JAK3/STAT3 signaling on c-KIT^+^ BM cells after 4 weeks. (**A**) Western blot showing increased phosphorylation of JAK3 (Y980/981) and STAT3 (Y705) among the c-KIT^+^ *MLL-AF9 knock in* cells in BM, after 4 weeks of HFD. Data show mean ± SD; n = 3 mice per diet group; ***P < 0.001, two-tailed unpaired Student’s t-test; ns, non-significant. Grouping of blots cropped from different gels, see Supplementary Fig. S6 for full-length blots. Gel images were processed and analyzed for quantification (Fiji, NIH). (**B**) Western blot showing increased phosphorylation of FLT3 among the c-KIT^+^ *MLL-AF9 knock in* cells in BM, after 4-weeks of HFD. After immunoprecipitation of pan tyrosine phosphorylated proteins with an anti-phosphotyrosine antibody (IP: pan Y), FLT3 was analyzed by a western blot (WB: FLT3). Increased phosphorylation of FLT3 following HFD was confirmed by immunoprecipitation with an anti-FLT3 antibody (IP: FLT3) followed by a western blot to detect pan phosphorylation of FLT3 (WB: pan Y). Data show mean ± SD; n = 3 mice per diet group; ***P < 0.001, two-tailed unpaired Student’s t-test. Grouping of blots cropped from different gels, see Supplementary Fig. S7 for full-length blots. Gel images were processed and analyzed for quantification (Fiji, NIH). (**C**) Flow cytometry on *MLL-AF9 knock in* BM cells showing median fluorescence intensity (MFI) for expression of FLT3 on Lin^−^ SCA1^+^ c-KIT^+^ (LSK) or Lin^−^ c-KIT^+^ (LK) cells. Data were analyzed using FlowJo software (version 10, TreeStar Inc, https://www.flowjo.com). RTqPCR, performed on cell-sorted cells, showing no modulation in transcription of the *Flt3* gene among LSK or LK cells, following a HFD. Data show mean ± SD; n = 4 mice per diet group; *P* value measured by two-tailed unpaired Student’s t-test; ns, non-significant. (**D**) Immunostaining showing that HFD induces cluster formation of lipid rafts (red), in which FLT3 (green) is typically condensed. Data on the left panel show examples of representative immunostaining for c-KIT^+^ cells from CD and HFD-fed mice. Microscopy is performed on c-KIT^+^ cells isolated by magnetic beads. Fluorescence was observed by microscopy (Axio Imager 2, Zeiss) and the images were processed (Fiji, NIH). White scale bar represents 5 µm. Arrows indicate FLT3/lipid raft clusters. Statistic is shown on the right panel. ***P < 0.001, two-tailed unpaired Student’s t-test.

Next, in order to elucidate the accelerated development of AML, we analyzed how a HFD affects signaling pathways on primitive HSPC isolated from BM. When we performed intracellular signaling by western blotting on primitive c-KIT^+^ cells collected from mice after 4 weeks of HFD, we discovered an increased activation of the JAK-STAT pathway, as characterized by an improved phosphorylation of JAK3 (*P* < 0.001) and STAT3 (*P* < 0.001). However, we observed no disturbance in AKT phosphorylation, and no phosphorylation was detected for STAT5 (Fig. 3A).

The FMS-like tyrosine kinase-3 (FLT3) activates the JAK-STAT pathway, which leads to increased proliferation/survival of human AML cells^18–22^. Moreover, MLL-AF9 was found to cooperate with activated FLT3 signaling to accelerate AML development in various mouse models^23–25^. Following a pull-down of all tyrosine-phosphorylated proteins, FLT3 was found to be highly phosphorylated (*P* < 0.001) in c-KIT^+^ cells isolated from HFD-fed mice, 4 weeks after the regimen. This increased phosphorylation of FLT3 was furthermore confirmed by immunoprecipitation with an anti-FLT3 antibody followed by western blotting to detect pan phosphorylation of FLT3 (*P* < 0.001) (Fig. 3B).

In conclusion, HFD activated the FLT3 receptor, which enhanced the downstream JAK3/STAT3 signaling among c-KIT^+^ cells.

### HFD increases clusterization of the FLT3 receptor within lipid rafts

As measured by flow cytometry, enhanced phosphorylation of FLT3 was not due to an increased expression of the FLT3 protein on the cell surface of the primitive HSPC from HFD-fed mice. In addition, we found no changes in *Flt3* gene transcription in these cells (Fig. 3C). As platforms for membrane trafficking and signal transduction, lipid rafts are master regulators of cytokine function, and they regulate several important key receptors involved in hematopoiesis^10,26–31^. Lipid rafts are cholesterol-enriched patches located in the plasma membrane, and the dynamic protein assembly in these lipid rafts can be modified by a disturbance in the lipid composition of cells^10,28^. Through immunostaining, we discovered that, after only 4 weeks of a HFD regimen, the localization of FLT3 within lipid rafts changed on the cell surface of c-KIT^+^ cells isolated from BM. We detected that the number of cells showing FLT3/lipid rafts clusters increased markedly (*P* < 0.001) among the population of c-KIT^+^ cells from HFD-fed mice (Fig. 3D). After the mice had consumed the HFD for 4 weeks, increased phosphorylation of FLT3 was detected in primitive HSPC, but few weeks after the diet had stopped, while the HFD was withdrawn and mice were fed a CD until they developed AML, this phosphorylation was no longer observed in post-HFD AML cells (Supplementary Fig. S4A–B). Indeed, immunostaining did not show colocalization of FLT3 among lipid rafts, and no clusters were visualized on post-HFD AML cells (Supplementary Fig. S4C).

We can therefore conclude that a HFD regimen can transiently modify clusterization of the FLT3 receptor within lipid rafts on c-KIT^+^ cells.

### Inhibition of the FLT3 phosphorylation blocks the HFD-enhanced development of AML

**Figure 4:**
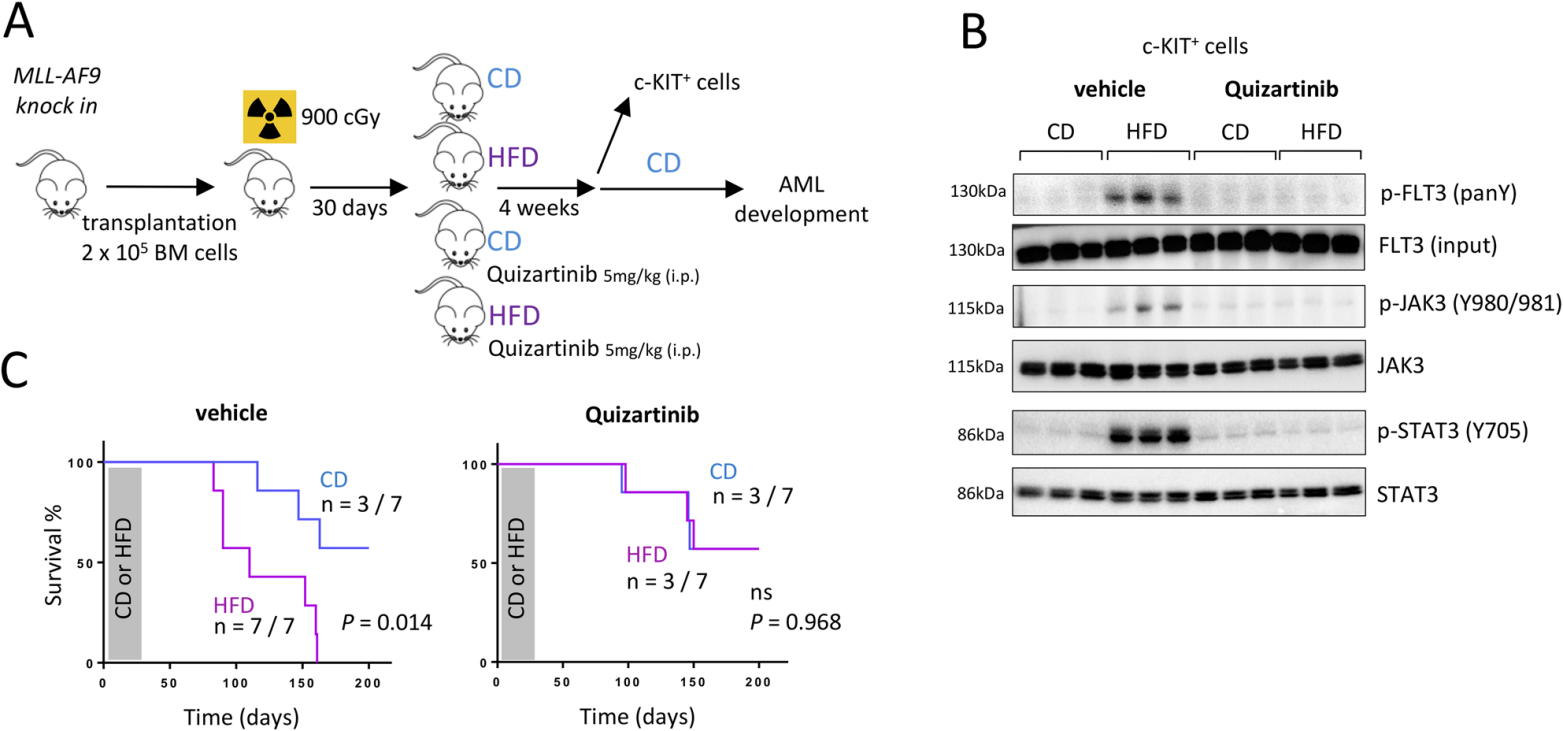
Quizartinib blocks the HFD-accelerated development of AML. (**A**) Experimental workflow describing the procedure. Image performed with the GIMP software (version 2.10.18, GIMP, https://www.gimp.org/news/2020/02/24/gimp-2-10-18-released/). (**B**) Western blot showing that a treatment with Quizartinib antagonizes the increased phosphorylation of FLT3 (pan tyrosine; panY) as well as JAK3 (Y980/981) and STAT3 (Y705) observed among the c-KIT^+^ *MLL-AF9 knock in* BM cells after 4-weeks of HFD. Data show mean ± SD; n = 3 mice per diet group. Grouping of blots cropped from different gels, see Supplementary Fig. S8 for full-length blots. Gel images were processed and analyzed for quantification (Fiji, NIH). (**C**) Survival curves showing that mice fed a HFD and treated with Quizartinib survived longer than CD-fed mice, n = 7 mice per group; *P* value measured by Mantel–Haenszel test.

To confirm involvement of FLT3/JAK3/STAT3 signaling in the accelerated transformation of *MLL-AF9 knock-in* cells, we injected Quizartinib, a potent inhibitor of FLT3 tyrosine phosphorylation^32–34^, showing efficiency between 1 to 10 mg/kg in vivo^34^. An intraperitoneal injection of 5 mg/kg was administered twice per week throughout the 4 weeks of the HFD (Fig. 4A). Four weeks after the beginning of the HFD, primitive HSPC expressed high phosphorylation/activation of the FLT3 receptor and showed active JAK/STAT signaling pathway. At the same time, c-KIT^+^ cells from mice that consumed the HFD and were treated with Quizartinib showed no activation of this pathway (Fig. 4B). When we examined mice for AML occurrence, HFD-fed mice developed AML much more quickly than did mice from the control group (*P* = 0.014). Meanwhile, HFD-fed mice treated with Quizartinib did not show an increased risk of developing AML (*P* = 0.968, Fig. 4C).

We can thus assume that the accelerated development of AML observed after consumption of a HFD is due to the increased activation of the FLT3 pathway.

## Discussion

Several mouse models have been described in the literature to study the role of HFD and leukemias and all studies show that HFD accelerates progression and aggressiveness of the diseases. Following a HFD feeding on a mouse xenograft model to study acute leukemia, alteration of the metabolism has been shown in the tumor microenvironment, which has significantly impacted the leukemia development^35^. Growth of xenotransplanted myeloid leukemia cell lines was also influenced by HFD in vivo^36^. Using two murine models, HFD-induced obesity has been shown to accelerate acute lymphoblastic leukemia progression^37^. The AML burden was also found much higher in HFD-induced obese mice, and the mechanism which link obesity with aggressive AML was due to an enhanced aberrant DNA methylation in AML cells^7,8^. All these findings, on several murine models used to study leukemias described more the role that obesity has on the tumor progression. In our study, we described for the first time, on a well-known murine model to study AML, that a HFD can accelerate the initiation of the *MLL-AF9*-driven AML disease. Furthermore, accelerated progression of AML has been described related to further mechanism, such as epigenetic which activate cell cycling^7,8^, changes in the metabolism of leukemic cells^36,37^, or in the microenvironment where cancer cells are expanding^35^. In our study, we described another mechanism, by which a HFD can modify the FLT3 signaling pathway known to accelerate the AML development in *MLL-AF9 knock-in* mouse model^24^. This mechanism involves that the HFD increases the clusterization of the FLT3 receptor within lipid rafts on the cell surface of primitive HSPC, which activates the phosphorylation of the FLT3 receptor, and can in turn triggers the stimulation of the downstream JAK3/STAT3 pathway in these cells.

FLT3 can be constitutively activated in human AML samples following either DNA duplication or mutations in the *FLT3* gene, which produce altered signaling^38–41^. Activated signaling in these models were consecutive to genetic modifications, such as expression of internal tandem duplications (ITD) or mutations in *FLT3*. On DNA extracted from post-HFD AML samples (n = 8 samples analyzed), we found no mutation or ITD in exons 14–15 of the *Flt3* gene encoding the juxtamembrane domain, and no mutation were found in exon 20 encoding the tyrosine kinase domain (TKD) in the *Flt3* gene (Supplementary Fig. S5). Therefore, the increased phosphorylation/activation of the FLT3 receptors and activated JAK/STAT signaling observed among primitive HSPC c-KIT^+^ cells isolated from mice after 4 weeks of HFD cannot be due to a genetic alteration of the murine *Flt3* gene.

While STAT3 and STAT5 proteins were both oncogenic downstream mediators of the JAK/STAT pathway^42^, STAT5 was actually shown as the target of constitutively active FLT3-ITD mutants, but not of the ligand-stimulated FLT3 wild-type receptor^43,44^. In our study, while no genetic alteration was detected in the *Flt3* gene, HFD overactivated the wild-type FLT3 receptor, and therefore only phosphorylation of STAT3 was found activated and no phosphorylation was detected for STAT5.

In animal model to study FLT3-ITD, Quizartinib has been described as a potent and selective inhibitor of FLT3 to treat mice bearing human AML xenografts^34^, in which there is a constitutive phosphorylation of FLT3 due to the ITD. In our study focusing on leukemia initiation, we have transiently fed mice a HFD, therefore active phosphorylation of the wild-type FLT3 receptor was observed following only the 4 weeks of HFD. This result highlights that HFD, even during a short period, can turn on the phosphorylation of FLT3, which increases the initiation of *MLL-AF9*-induced AML. While after this regimen mice were fed a CD until they developed AML, there was no active phosphorylation of FLT3 observed in post-HFD AML samples (Supplementary Fig. S4B). Indeed, it does not seem judicious to us to treat post-HFD AML mice with Quizartinib, due to the absence of overactivation of FLT3 in post-HFD AML, this treatment should not be able to reduce the leukemia progression.

## Conclusion

In summary, we show for the first time that a HFD can overactivate the FLT3 receptor on primitive hematopoietic cells, as well as the downstream JAK/STAT signaling known to accelerate transformation of *MLL-AF9 knock-in* BM cells. Treatment of mice with Quizartinib inhibited FLT3 phosphorylation on primitive hematopoietic cells, which resulted in the blockade of the JAK3 and STAT3 phosphorylation that typically occurred during the HFD regimen, and finally antagonized the initiation of AML and its accelerated development. We can therefore conclude that a HFD regimen enforced the expression of FLT3 signaling and enhanced the oncogenic transformation of *MLL-AF9 knock-in* BM cells. Using a mouse model of AML, we revealed that consuming a HFD, even in the short term, accelerates the risk of leukemia development.

## Supplementary information

Supplementary Figures.
