# Supplementary material for: High-fat diet intensifies MLL-AF9-induced acute myeloid leukemia through activation of the FLT3 signaling in mouse primitive hematopoietic cells
Source: Sci Rep. 2020 Sep 30;10:16187. doi: 10.1038/s41598-020-73020-4 (PMC7528010; doi:10.1038/s41598-020-73020-4)
Supplement: Supplementary file 1 — Supplementary Figures. [file 41598_2020_73020_MOESM1_ESM.pdf]

High-fat diet intensifies *MLL-AF9*-induced acute myeloid leukemia through activation of the FLT3 signaling in mouse primitive hematopoietic cells

François Hermetet,[1,2,*] Rony Mshaik,[1,*] John Simonet,[1] Patrick Callier,[3] Laurent Delva[1,2] and Ronan Quéré[1,2]

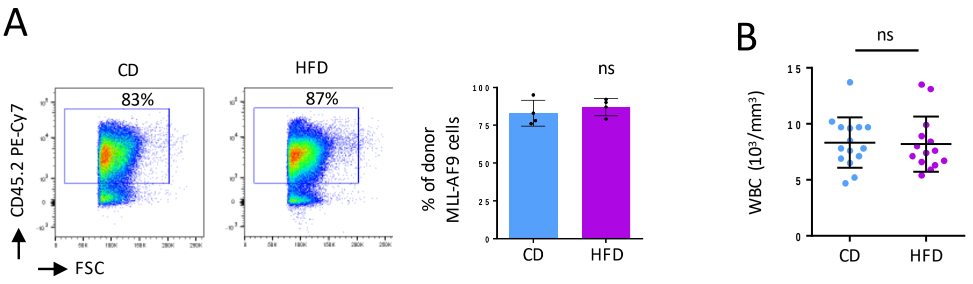

Figure S1. HFD did not alter engraftment of *MLL-AF9 knock-in* Ly.2 cells to the BM, as well as reconstitution of total white blood cells (WBC) in PB of recipient mice. (**A**) Flow cytometry showing similar engraftment of *MLL-AF9 knock in* cells (CD45.2 positive cells) in the BM of mice fed with either a CD or a HFD. Experiment done on the BM, after 4 weeks of CD or HFD, n=4 mice per diet group. Data were analyzed using FlowJo (version 10, TreeStar Inc, https://www.flowjo.com/, under the license number S13Q5La2Ft1g6ARY). (**B**) After 4 weeks, HFD did not alter the reconstitution of WBC in PB of recipient mice, compared with CD. One mouse from the HFD group had developed AML, after 4 weeks (see **Fig. 1B**) and was therefore not included in this analysis. Data show mean ± SD; n=15 and 14 mice for CD and HFD groups, respectively; *P* value measured by two-tailed unpaired Student's t-test; ns, non-significant.

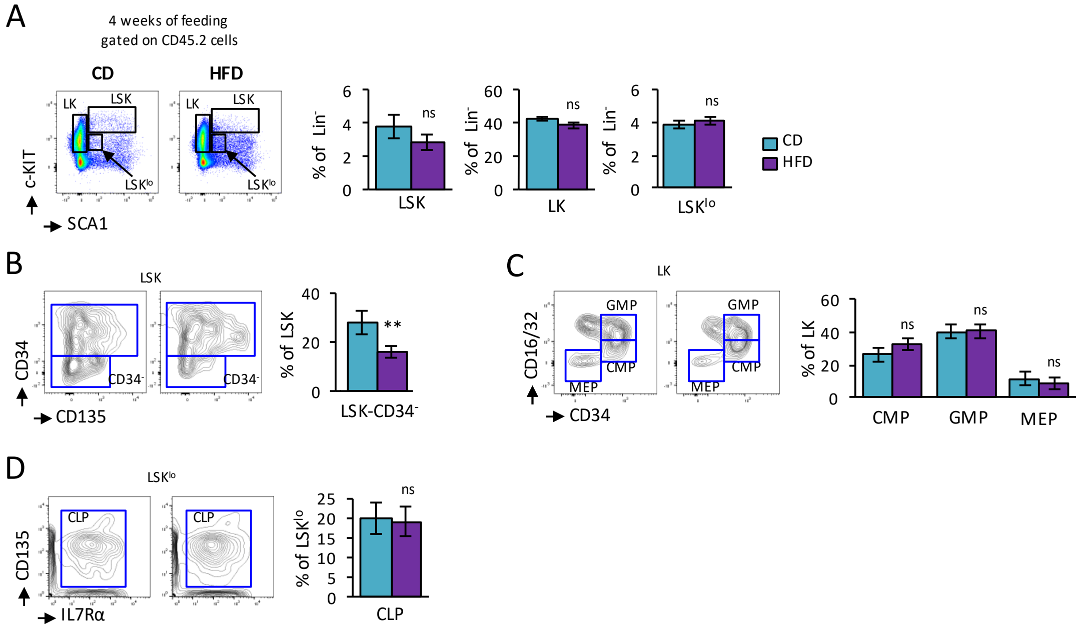

Figure S2. Flow cytometry analyses showing the main changes observed among the populations of primitive HSPC following a HFD. (**A**) Flow cytometry analysis on lineage negative (Lin^{-}) SCA1^{+} c-KIT^{+} (LSK), Lin^{-} SCA1^{-} c-KIT^{+} (LK) and Lin^{-} SCA1^{+} c-KIT^{lo} (LSK^{lo}) cells. (**B**) Flow cytometry analysis showing a decreased number of primitive HSC (LSK CD34^{-}) among LSK cells from HFD-fed mice compared to CD-fed mice. (**C**) Flow cytometry analysis on LK showing no variation in the number of mature progenitors such as mega-erythroid progenitor (MEP), common myeloid progenitor (CMP), granulocyte/macrophage progenitor (GMP). (**D**) No variability is furthermore observed among the common lymphoid progenitor (CLP) following a HFD feeding. On this figure, flow cytometry analysis was performed on Lin^{-} CD45.2^{+} *MLL-AF9 knock-in* BM cells, after depletion of Lin^{+} BM cells, and after 4 weeks of feeding mice with CD (n=3 mice) or HFD (n=3 mice). Data were analyzed using FlowJo software (version 10, TreeStar Inc, https://www.flowjo.com/, under the license number S13Q5La2Ft1g6ARY). Data show mean

± SD; *P* value measured by two-tailed unpaired Student's t-test; **, *P*<0.01; ns, non-significant.

A

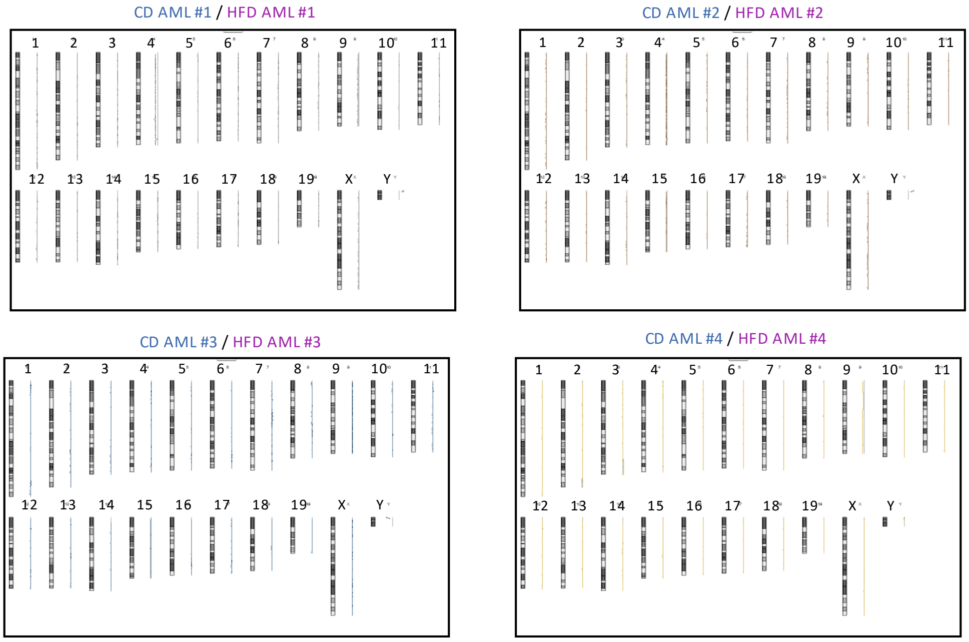

B

Aberration No	Chr	Cytoband	Start	Stop	#Probes	Amplification	Deletion	pval	Gene Names
CD AML #1 / HFD AML #1									
1	chr4	qA1 - qE2	3159538	155607029	9972	0	-0,4948	0.000	Tmem68 to Klhl17
2	chr4	qD1	111726014	113338225	112	0	-0,810532	5.004e-54	Skint4 to Skint6
3	chrY	qA1	66224	1551431	95	0,678196	0	1.522e-55	Zfy1 to Zfy2
CD AML #2 / HFD AML #2									
1	chrY	qA1	57466	2506368	112	0,604967	0	1.247e-79	Zfy1 to Rbmy1a1
CD AML #3 / HFD AML #3									
1	chr1	qD	90115633	90193738	10	0,564639	0	1.061e-17	Ugt1a10 to A730008H23Rik
2	chr7	qA3	26790284	26981899	8	0	-0,570104	1.379e-14	Cyp2b13 to Cyp2b9
3	chr7	qB4	54747626	55264057	17	0,462549	0	9.301e-14	Mrgpra3 to Mrgpra4
4	chr7	qC	67341095	67731725	16	0,679526	0	9.055e-29	
5	chr7	qE3	110691909	110709040	3	0,875214	0	2.460e-10	Olfr614 to Olfr615
6	chr11	qB4	70989862	71107103	11	0	-0,620221	2.128e-22	Nlrp1b to Nlrp1c
7	chr11	qE2	116606695	116626075	3	1,648338	0	1.484e-29	BC018473
8	chr13	qB3	61743712	62048006	7	0,63084	0	2.443e-15	
9	chrY	qA1	249096	1610834	95	0	-0,775322	3.485e-97	Kdm5d to Zfy2
CD AML #4 / HFD AML #4									
1	chr9	qA5.3 - qF4	49325230	124038609	5509	0	-0,25189	1.479e-51	Ncam1to Ccr5

Figure S3. CGH-array analysis showing that there was no significant genetic alteration in DNA isolated from post-HFD AML cells when compared with post-CD AML cell samples. CGH-arrays were performed on 4 DNA (#1-4) isolated from post-CD and HFD AML. (**A**) Chromosomes of the CGH-arrays. Images were processed using the Agilent Cytogenomics software (version 2.7, Agilent Technologies, https://www.agilent.com/en/download-agilent-cytogenomics-software). (**B**) Main genetic aberrations observed by CGH-array analysis.

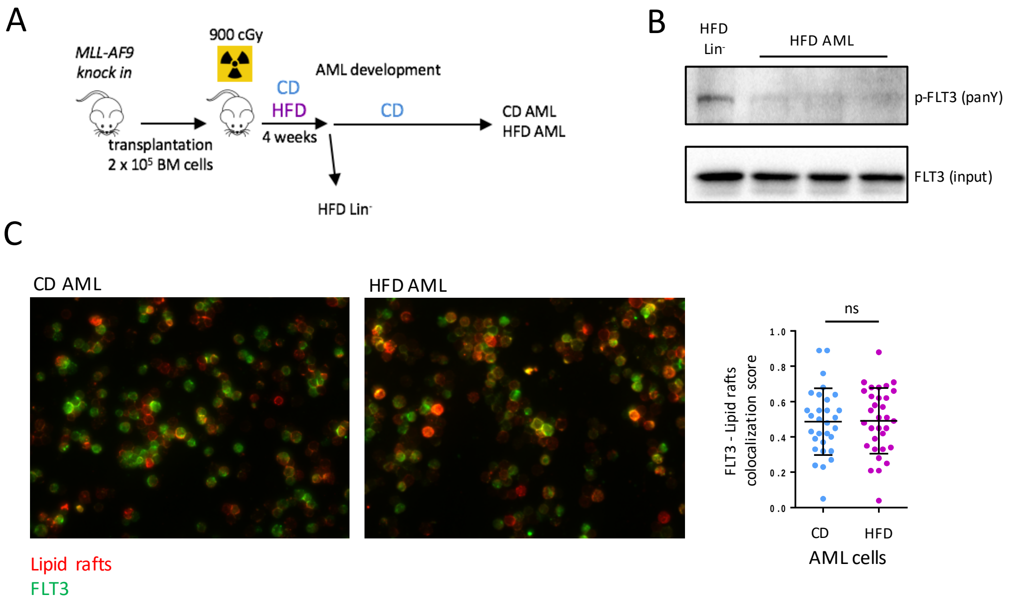

Figure S4. Experiments displaying that AML cells are not showing activation of FLT3. (**A**) Experimental workflow describing the procedure. Image performed with the GIMP software (version 2.10.18, GIMP, https://www.gimp.org/news/2020/02/24/gimp-2-10-18-released/). To obtain post-HFD AML samples, HFD was only provided to the mice for 4 weeks and was replaced by CD several weeks before the occurrence of AML. (**B**) Western blot showing no phosphorylation (pan tyrosine; pan Y) of FLT3 among post-HFD AML cells (HFD AML) isolated from 3 mice, compared with Lin$^-$ cells isolated from a mouse fed with a HFD during 4 weeks and sacrificed just after the HFD (HFD Lin$^-$). Grouping of blots cropped from different gels, see Supplementary **Fig. S9** for full-length blots. Images were processed using Fiji software (NIH). (**C**) Microscopy showing no colocalization between lipid rafts (stained with AF555-conjugated cholera toxin subunit B, red) and FLT3 (stained with anti-FLT3 antibody and anti-rabbit-AF488 antibody, green). Representative immunostaining for post-CD and HFD AML cells is shown on the left. Images were obtained using Axio Imager 2 (Zeiss) and the images were processed with Fiji software (NIH). Graphic on the right showing the colocalization score between lipid rafts and FLT3 on single cells (n > 30 cells). *P* value measured by two-tailed unpaired Student's t-test; ns, non-significant.

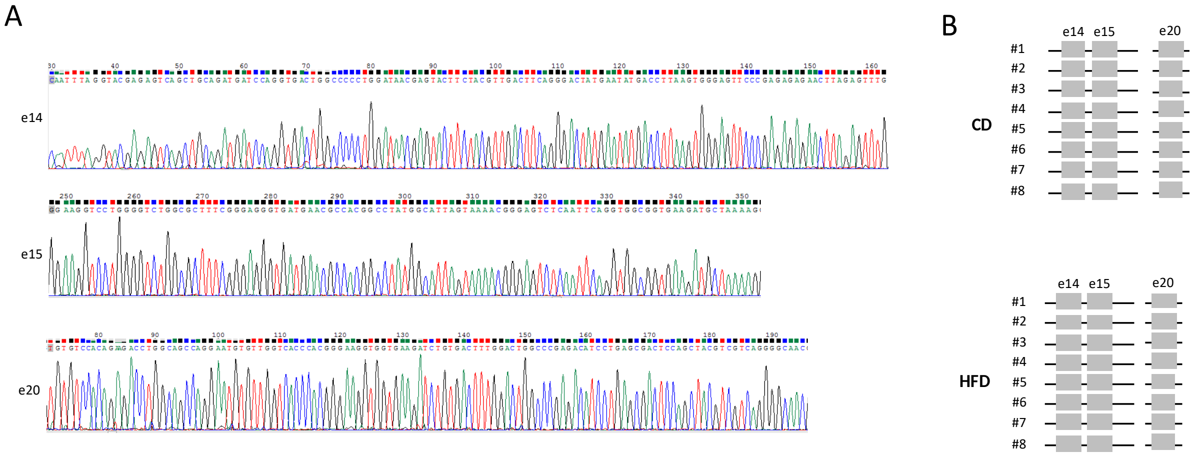

Figure S5. On DNA extracted from post-CD and -HFD AML cells (n=8 samples analyzed per group), no mutation or duplication were observed in exon 14 (e14), exon 15 (e15) and exon 20 (e20) of the *Flt3* gene. (**A**) Data showing representative result of Sanger sequencing performed on one post-HFD AML sample; no heterozygote mutation was detected. Images were created using Chromas (2.6.6) software (Technelysium, http://technelysium.com.au/wp/chromas/). (**B**) Sanger sequencing performed on DNA purified from 8 post-CD and 8 post-HFD AML cells (#1-8). Grey square is for DNA from BM cells of wild type mouse, without any heterozygote mutation detected by Sanger sequencing.

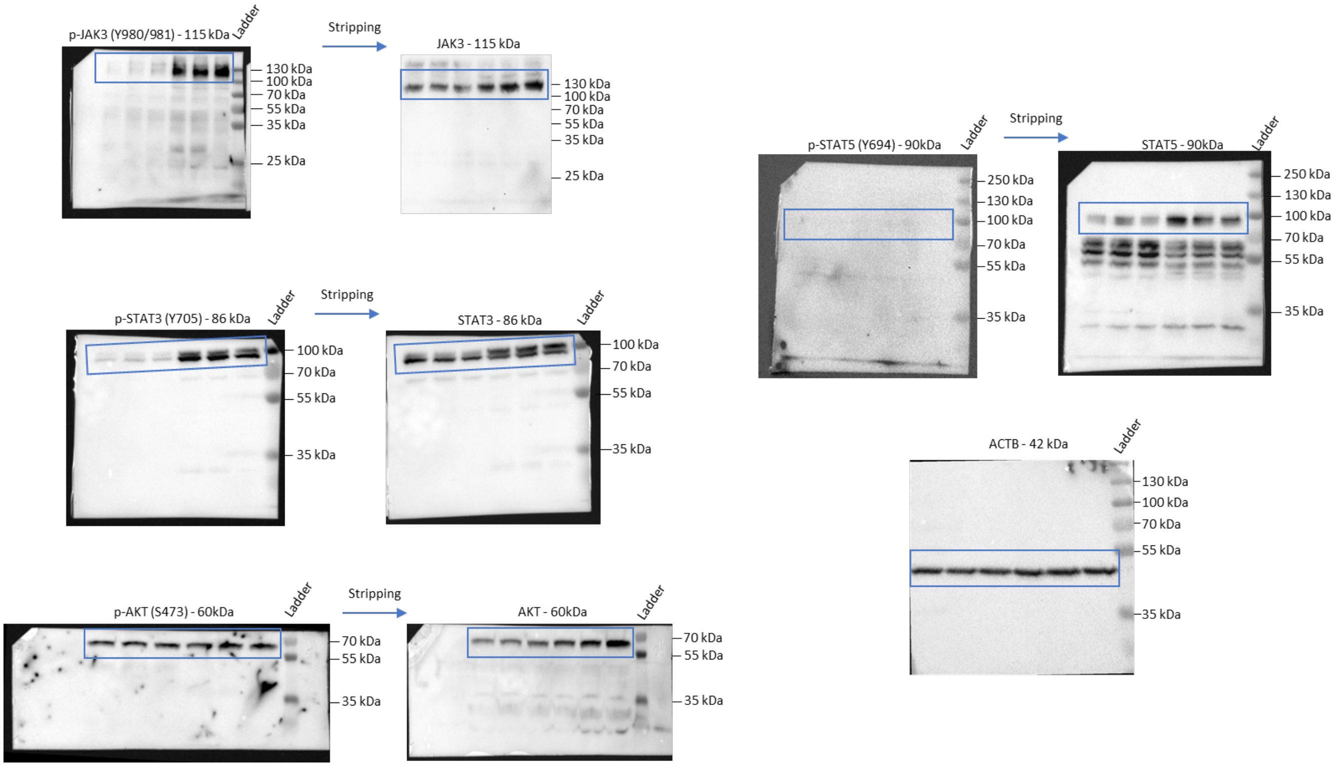

Figure S6. Full-length blots corresponding to Fig. 3A. For the p-AKT and AKT blots, acrylamide gels were cut prior to transfer on PVDF membranes.

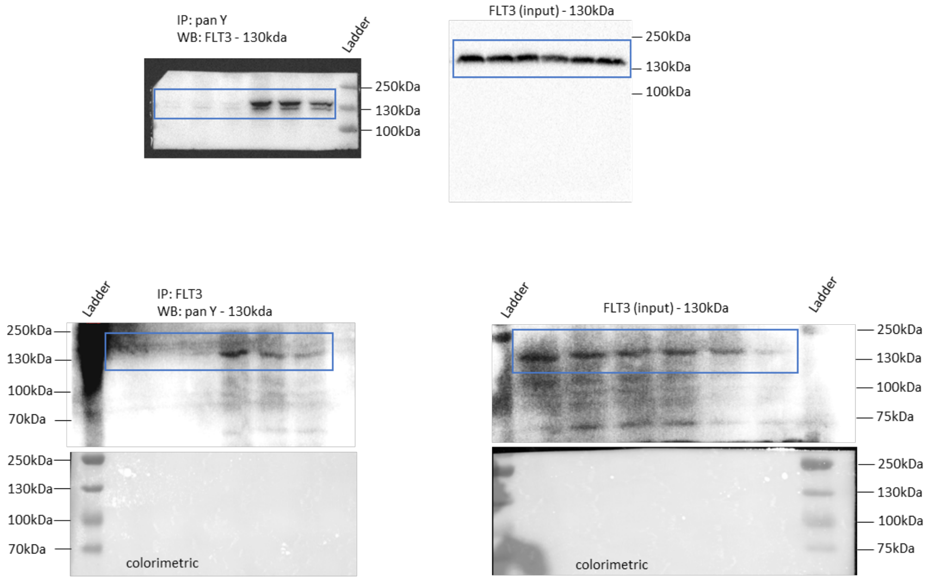

Figure S7. Full-length blots corresponding to Fig. 3B. For the blots, acrylamide gels were cut prior to transfer on PVDF membranes.

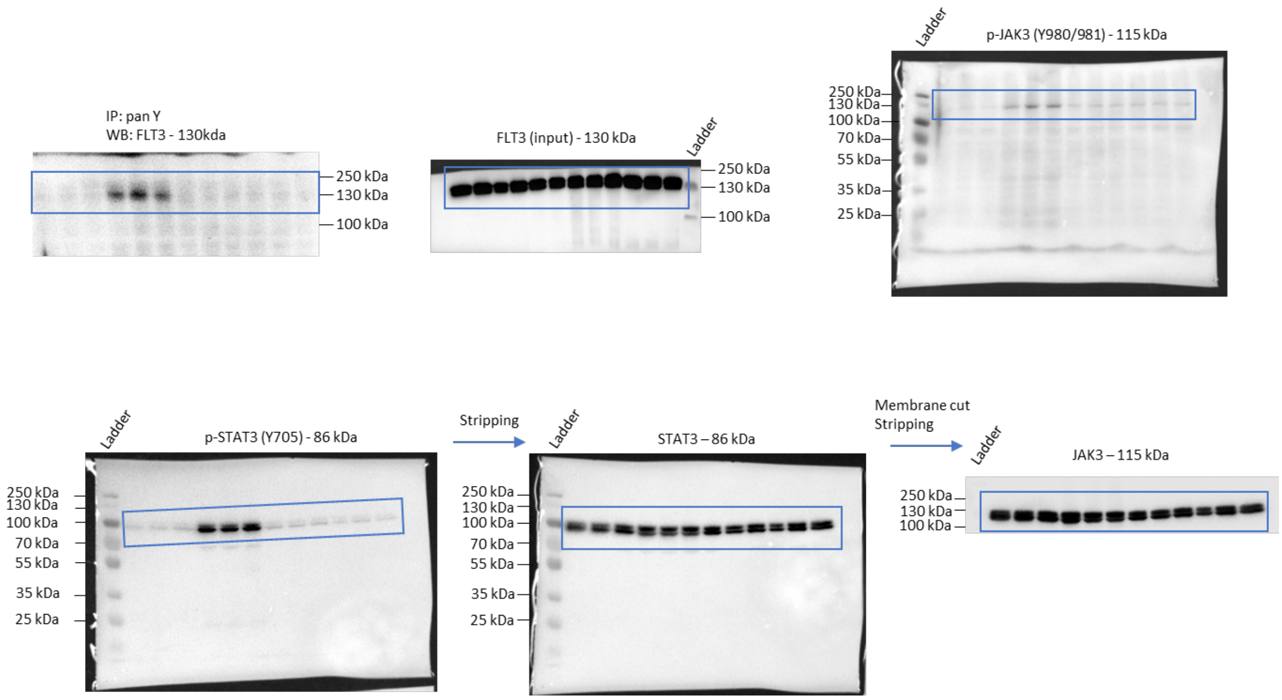

Figure S8. Full-length blots corresponding to Fig. 4B. For the FLT3 blots, acrylamide gels were cut prior to transfer on PVDF membranes. For the JAK3 blot, the membrane was cut prior to staining with the anti-JAK3 antibody.

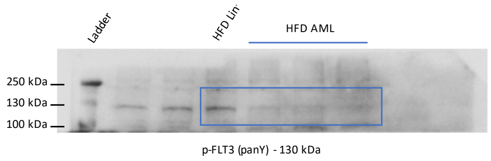

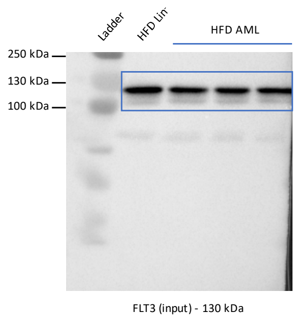

Figure S9. Full-length blots corresponding to Supplementary Fig. S4. For the p-FLT3 blot, acrylamide gel was cut prior to transfer on PVDF membrane.
